# Lived experiences of nurse educators on teaching in a large class at a nursing college in Gauteng

**DOI:** 10.4102/curationis.v39i1.1507

**Published:** 2016-07-08

**Authors:** Maria G. Ndawo

**Affiliations:** 1 Nursing Department, University of Johannesburg, South Africa

## Abstract

**Background:**

The gradual increase in the number of learners admitted into a nursing college in Gauteng resulted in an increase in class size without a proportional increase in the number of nurse educators.

**Objectives:**

To explore and describe the experiences of nurse educators teaching in large classes at a nursing college in Gauteng in order to present recommendations to facilitate teaching and learning.

**Method:**

A qualitative, exploratory, descriptive, and phenomenological research design which is contextual in nature was used. A total of 20 nurse educators were selected through purposive sampling, and in-depth phenomenological semi-structured individual interviews were conducted between January and February 2013. Data were analysed together with the field notes, using Tesch’s open coding protocol of qualitative data analysis. Lincoln and Guba’s four principles were used to ensure trustworthiness.

**Results:**

The themes that emerged from this study were that nurse educators experienced difficulty in recognising learners as individuals in a large class, using innovative pedagogical strategies, and managing a large class. These findings had a negative impact on meaningful teaching and learning as they interfered with an enabling learning environment.

**Recommendations:**

Nurse educators should be empowered with facilitative skills in order to effectively manage a large class and hence to achieve teaching and learning abilities.

**Conclusion:**

There is a need for nurse educators to finding alternative ways to overcome challenges associated with teaching in large classes and prepare learners to render individualised, caring and holistic nursing care to each unique patient in the healthcare setting.

## Introduction

There has been a gradual increase in the number of learners admitted into nursing colleges nationally, which has resulted in increased class sizes. The Gauteng Department of Health (DoH) had prescribed a 15% to 20% annual increase in the number of learners in its nursing colleges since 2004 (South African Government 2011). However, this has not been accompanied by the proportional increase in the number of nurse educators that would be needed under the circumstances (DoH 2013:21). In 2004, the enrolment figure of learners in a first year class at the nursing college under study was 213 whereas in 2012 it was 367 which is a 58% increase in enrolment figures over 9 years. The first year class size increased to about 500 learners at the time of the study. The increase is further exacerbated by the high number of repeaters resulting from high failure rate amongst first year learners (DoH 2013:28). The current class size is not in keeping with the prescripts of nurse educator to learner ratio, namely 1:15–20 (DoH 2013:85). In the nursing college which is the site for the study, the nurse educator to learner ratio was 1:150 for most of the first year subjects such as sociology, fundamental nursing science and general nursing science 100.

In large classes, benefits such as diversity of accumulative knowledge, skills, experiences, interests and ideas enliven discussions amongst learners. Learners share responsibility for learning that facilitates meaningful collaborative and cooperative interaction. They learn that there are multiple solutions to a problem, hence, they develop intellectually and become lifelong, self-directed learners (Kerr 2011:8). Nurse educators have opportunities to adopt and implement technology-driven and innovative pedagogical strategies (Wanous, Procter & Murshid 2009:79). The success of using these pedagogical strategies is evident in New Zealand where the national teaching excellence awards are won by educators of classes of 850 learners (Exeter *et al*. 2010:762). Many studies show evidence that innovative pedagogical strategies can lead to effective learning and development of higher order thinking skills even in large classes such as those cited by Abu Hasheesh, Al-Mostafa and Obeidat (2011) from 1996 to 2010. However, these benefits should be viewed bearing the anecdotal disadvantages in mind.

In spite of the acknowledged benefits of teaching in a large class, for most nurse educators it remains a real challenge. Extreme diversity in age, academic experience, cultural background and socio-economic status is, on the other hand, viewed to pose a challenge and hinder active participation (Mulryan-Kyne 2010:176). The nurse educators often have the challenge of teaching learners who are academically underprepared (Oliver 2012:4). In large classes the challenge becomes worse as the nurse educators experience difficulties in identifying individual learning needs. In large classes learners experience an impersonal learner-educator environment that results in anonymity, passivity, lack of individual accountability, frustration, and educators being unable to relate to them as individuals (Gosselin 2012:238). The learner experiences then lead to high levels of absenteeism because learners know that nurse educators do not know them. Moreover, large classes limit the chances to provide academic advice, one-on-one remediation and guidance to learners who are struggling academically (Brett, Branstetter & Wagner 2014:362).

In large classes, nurse educators experience difficulties in adopting and using the technology-driven and innovative pedagogical approaches to learning which develop higher order thinking skills in learners; approaches such as problem-based learning (PBL), dialogic pedagogy, reflective learning, chatrooms, blackboard and videoconferencing (Kerr 2011:17; Ndawo 2014:154). Nurse educators rely predominantly on the lecture method to deliver learning content, which limits learners’ interaction in class resulting in disengagement of learners with the learning content inside and outside of the classroom. As early as the 1960s, Rogers (in Regmi 2012:67) approached education in a way that saw each learner as an individual with unique characteristics, and who is capable of self-awareness and self-evaluation even in a large class. In an enabling learning environment, each learner should be viewed as an individual to be valued, cared for, respected, nurtured, understood and assisted. Therefore, the concern for the individuality of each learner is important. In a large class, Rogers’s approach is not realised because of high numbers of learners rendering the learning environment not enabling (Ndawo 2014:91).

### Problem statement

Nurse educators are challenged to meet the required learning outcomes of the nursing programme such as facilitation of critical thinking, problem-solving, rational decision-making and astute clinical judgment as a result of large classes. In the first year of study, learners are still adapting to the tertiary institution environment, yet are immersed in large classes. In a large class there is anonymity, lack of discipline, difficulty to manage learners, difficulty in utilising innovative pedagogical strategies that develop higher order thinking skills in learners, and a lack of guided facilitation by nurse educators (Kerr 2011:8; Mulryan-Kyne 2010:176). The compounding challenges may lower the academic performance of nurse educators, resulting in learner underachievement, academic failure and a decreased output of nurses (Gosselin 2012:238). Nurse educators are expected to keep abreast of recent advances in pedagogical approaches and new technologies to improve the large class experience (Kerr 2011:9). However, in the present study, nurse educators as participants, experienced difficulties teaching in large classes of first year learners. Thus, the study seeks to explore and describe the experiences of nurse educators regarding teaching in a large class at a nursing college in Gauteng in order to present recommendations to facilitate effective teaching and meaningful learning.

The research questions that emerged were:

- What are the experiences of nurse educators regarding teaching in a large class at a nursing college in Gauteng?
- What can be done to facilitate effective teaching and meaningful learning in a large class at a nursing college?

#### Research aim

The aim of the study is to enhance teaching through presenting the recommendations to assist nurse educators teaching in large classes at a nursing college in Gauteng.

#### Research objectives

The research objectives that guided this study were:

- To explore and describe the experiences of nurse educators regarding teaching in a large class at a nursing college in Gauteng.
- To describe recommendations to facilitate effective teaching and meaningful learning in a large class.

#### Definitions of key concepts

**Large class** is one in which nurse educators can no longer cope with the number of learners even if they have a desire to provide individual attention to them (Tripod n.d.:2). In this study a large class refers to a class of 500 learners in the first year of study.

**Experience** is the knowledge that comes from being personally involved in an event, situation, or circumstance (Burns & Grove 2009:9). In this study, experience relates to the personal knowledge that nurse educators possess of teaching in a large class.

**Nurse educator** is a person who has the ability to communicate knowledge to learners, diagnose learners’ educational needs, formulate strategies to facilitate learning in various ways and evaluate learning for the purpose of developing the learners’ reflexive skills (Bruce, Klopper & Mellish 2011:108). In this study, a nurse educator is a person who taught and/or teach about 500 first year learners at a given time.

**Learner nurse** is a person undergoing education and training in nursing and must be registered as such by the South African Nursing Council (SANC) as stipulated in the *Nursing Act* (Act no. 33 of 2005). In this study, a learner nurse will be referred to as a learner.

**Teaching** is a deliberate and purposeful activity directed towards the promotion of learning that comprises two basic elements, namely the teacher who is doing the promoting of learning and the learner whose learning is thus promoted (Bruce *et al*. 2011:21).

**Nursing college** is a post-secondary education institution that offers professional nursing education at basic and post-basic level where such nursing education has been approved in terms of section 15(2) of the *Nursing Act* (Act no. 33 of 2005). In this study the nursing college referred to is situated in Gauteng.

### Contribution to field: Significance of the study

The findings of this study highlighted that a class of 500 learners was experienced as a hindrance by the nurse educators, and recommendations were consequently made with regard to how teaching and learning in large classes can be effectively and meaningfully facilitated at a nursing college.

## Research design and method

### Design

A qualitative, exploratory, descriptive, and phenomenological research design which is contextual in nature was used (De Vos *et al*. 2011:95; LoBiondo-Wood & Haber 2014:109). Qualitative research is a ‘… systematic, interactive subjective approach used to describe lived experiences and give them meaning’ (Burns & Grove 2009:51). The researcher sought to explore the depth, richness and complexity inherent in the lived experiences of nurse educators who teach large classes at a nursing college. A hermeneutic, interpretative phenomenological method was used in this study to capture the essence of the lived experiences of participants (Walker 2011:19). A hermeneutic method focuses on understanding the phenomenon in-depth, more than just describing it. The researcher explored, as well as interpreted the meaning of nurse educators’ lived experiences concerning teaching in a large class.

Phenomenological studies focus on exploring the participants’ lived experiences as they occur or had occurred in the past. This is important as phenomenologists believe that each individual’s past is part of the present (LoBiondo-Wood & Haber 2014:112). The use of the phenomenological method was facilitated through bracketing and intuition (Burns & Grove 2009:545). Bracketing was performed to ensure neutrality and demonstration of truth value of the findings, thus preventing the researcher from imposing preconceived ideas, assumptions or biases on the study. Intuition was aimed at creating a rich and deep account of the experiences of the participants through an attempt to exclude the researcher’s prior knowledge and beliefs about the phenomenon (Kafle 2011:182, 190).

### Population and sampling

In phenomenological studies, participants are selected through the use of a non-probability, purposive sampling method (Burns & Grove 2009:355). A purposive sampling method was used in order to select participants who could provide rich and in-depth data regarding teaching in a large class. From these participants, the researcher learnt a great deal about the phenomenon under study. The accessible population of this study consisted of 104 nurse educators who taught or are still teaching first year learners. Learners were registered for the 4 year comprehensive diploma programme leading to registration as a nurse (general, psychiatric and community) and midwife as stipulated by the SANC Regulation 425 of 1985 (as amended) at a nursing college in Gauteng. Participants were selected based on the following inclusion criteria: nurse educators registered with SANC under Regulation 118 of 1987 (SANC 1987), who teach and/or taught learners of more than 450 in a class at one specific given time. Nurse educators must have taught in a large class of first year learners in the last 2 years. Nurse educators who were willing, volunteered and consented to participate in the study. Data were collected until saturation was achieved at the 20th participant.

### Data collection

Data were collected by the researcher using phenomenological semi-structured individual interviews in order to allow participants maximum opportunity to share their lived experiences freely (De Vos *et al*. 2011:296). The phenomenological interviews were conducted in English between January and February 2013 during a practical block which ensured that teaching and learning activities were not interrupted. The duration of each interview was approximately 45 to 60 min. The date, time and venue was chosen in accordance with the participants’ preferences. The main research question that guided the phenomenological interviews was: ‘What are your experiences of teaching in a class of about 500 learners at a given time at this nursing college?’

The second question was: ‘What can be done to assist you facilitate effective teaching in this nursing college?’ The researcher assumed a neutral position during the phenomenological interviews through bracketing her own perceptions and beliefs about teaching in a large class in order to avoid bias (Burns & Grove 2009:545). Communication clarification techniques, such as probing, summarising, paraphrasing, listening, reflecting, using minimal verbal response and focusing (De Vos *et al*. 2011:289), were used to ensure detailed exploration of the experiences of nurse educators who teach in a large class. The phenomenological interviews were audio-taped with the permission of the participants to increase the credibility of the study by accurate capturing and transcription of the data. Field notes were taken to enrich the collected data. Data collected were transcribed verbatim as soon as possible after each interview and as soon as data collection was completed (De Vos *et al*. 2011:39).

### Data analysis

The data were analysed using Tesch’s open coding protocol of qualitative data analysis as described in Creswell (2012:64). Tesch’s data analysis method was chosen because it follows eight descriptive and logical steps of in-depth intricacy and meticulous analysis of qualitative data. The researcher listened to the audiotapes, read and re-read the verbatim transcripts of the phenomenological semi-structured individual interviews one at a time repeatedly in order to gain a global understanding of the interviews. Therefore, the researcher became immersed in the data. The most interesting interview was chosen and ideas and patterns pertaining to the lived experiences of nurse educators were underlined. The researcher then continued to read the rest of the interviews, identifying similar ideas and patterns. These similar ideas and patterns were coded and grouped together into themes. The data of each theme were assembled and a preliminary analysis of information and comparison between the different interviews were carried out. Existing data were recoded where necessary. To establish the credibility of the data, an independent coder who is an expert in qualitative data analysis was used to analyse the data independently. The researcher and the independent coder held a consensus discussion meeting to reach an agreement on the independently identified themes (Creswell 2013:253). Follow-up individual interviews with six participants were organised to verify the accuracy of the identified themes.

### Context of the study

The study was conducted at a nursing college in Gauteng amongst nurse educators who were once, or still are, involved in teaching between 450 and 500 first year learners. These learners were registered for the 4 year comprehensive diploma programme leading to registration as a nurse (general, psychiatric and community) and midwife as stipulated by SANC Regulation 425 of 1985 (as amended).

## Ethical considerations

Ethical decisions in this study were observed based on guidelines pertaining to the principle of respect for persons, beneficence and justice (Burns & Grove 2009:188). Informed consent to participate voluntarily in the study and the use of a tape recorder was obtained from all participants after explaining the purpose, objectives, methods and their expectations from the study. Pseudonyms were used to ensure anonymity, and confidentiality was ensured through the safe-keeping of the audio-taped interviews and transcriptions under lock and key. No one except the researcher and the independent coder had access to the collected data.

## Trustworthiness

The researcher adopted Lincoln and Guba’s (1985:290–327) four strategies of establishing trustworthiness of qualitative data, namely, credibility, transferability, dependability and confirmability. Credibility was ensured by spending sufficient time with nurse educators during data collection, ensuring triangulation through taking field notes, and using different primary and secondary sources. Reflexivity was achieved through a self-examination process during a discussion with the independent coder where reflection on the data was carried out in order to eliminate biases and interpret the truth. It was also achieved by the use of an audio-tape recorder and taking field notes, in which verbal and non-verbal observations were recorded as they occurred (Creswell 2013:216). Member checking was performed through six follow-up interviews with participants in order to verify the themes. Transferability was ensured by thick description of the research methodology, the research context, and purposive sampling. This was carried out in order to enable other researchers interested in the replication of the study. Dependability of the study was enhanced by providing a dense description of the research methodology. All the audio-taped raw material, transcripts and signed consent forms were kept under lock and key for the purposes of conducting an inquiry audit. A code-recode procedure was employed during data analysis. Confirmability was ensured by a confirmability audit trail and the use of an independent coder. The phenomenological semi-structured individual interviews were enriched with field notes and accuracy ensured by use of an audio-tape recorder (De Vos *et al*. 2011:346).

## Findings and discussions

The findings of this study revealed that there were no positive experiences indicated by the participants. The major themes that emerged were difficulty in recognising learners as individuals, using innovative pedagogical strategies, and managing a large class. The findings were conceptualised within relevant literature. Recommendations by participants were also conceptualised within the discussion.

### Difficulty in recognising learners as individuals in a large class

Most participants stated that they had difficulty in recognising learners as individuals in a large class. Learners were unknown to one another, as well as to the nurse educator, and this promoted learners’ disengagement with the nurse educators, peers and learning content. This is confirmed by the following comments from participants:

‘…The large number impacts on the type of class you are going to have because now you have … more than 450 learners in one class (frowning). It goes against the principles of teaching, you hardly know the learners, there’s no eye contact, you cannot read their faces … when they are getting confused, and when they have a puzzled look. Are they bored, are they listening, or are they lost?’ (P2)

‘…At any given time … you are teaching close to 500 students. How could you possibly know all these students? It’s difficult, actually it’s impossible for any human being to keep up with all those names and faces.’ (P19)

‘…As an educator you can’t pick up the ‘weaklings’ with these huge numbers. You only pick them up during your marking.’ (P8)

In a large class there are circumstances that present themselves as limitations that impede meaningful learning (Iipinge 2013:107). In this study learner numbers were too large for nurse educators to become acquainted with each learner on a personal and individual basis. Not knowing their learners, resulted in difficulties for nurse educators to facilitate effective teaching. Elder, Seaton and Swinney (2010:92) state that in a large class the ‘… learner becomes a nameless face in a sea of faces, gets lost in a crowd’, and that results in anonymity. Anonymity contributes to de-individualisation which is the ‘… process by which an individual’s personal identity is replaced by the identity of the group’ (Elder *et al*. 2010:93). In this class, the individual learners view their participation and actions as trivial and insignificant. As a result some learners have perceptions that no one knows them, no one notices them, so no one cares about them (Kerr 2011:8). This causes them to feel dissociated from each other and these feelings encourage absenteeism and reluctance toward studying.

It also emerged that, owing to large numbers of learners, most nurse educators had challenges in identifying and providing individual attention early to ‘at risk’ learners whom they referred to as ‘weaklings’. At risk learners are those who have a higher probability of failing academically or dropping out of a nursing programme (Pearson & Naug 2013:136). The larger the class of learners, the more at risk learners a nurse educator has to deal with. Of importance is that usually the academically at risk learners tend to seek help from other at risk learners resulting in a ‘blind-leading-the-blind’ situation. There are many factors that place learners at risk of academic failure; lack of nurse educator support was cited by Harris, Rosenberg and O’Rourke (2014:33) as one that was prevalent in a large class. By not diagnosing learners’ unique learning needs, they have limited opportunity to succeed academically.

When asked what can be effected to improve this situation, participants stated that:

‘… Closed nursing colleges should be reopened so that we have more colleges, smaller classes, and fewer students.’ (P8)

‘… Nursing colleges must have multiple intakes in a year … Multiple intakes decreases [*sic*] the number of students into manageable numbers and decrease workload.’ (P1)

‘… [*B*]efore students were divided into two groups of 250 each, but the challenge was that students had complaints about what was taught in alpha group was different to what was taught in beta group or they complained about the manner in which it was taught, stating that it was not the same.’ (P11)

### Difficulty in using innovative pedagogical strategies

Participants further reported that, because of large classes, they predominantly use the lecture method to teach learners. This ensured that the learning content was delivered to learners as well as the content covered; however, this method restricted the use of innovative pedagogical strategies. The following comments confirm this finding:

‘… The only comfortable method to use in a large class is the lecture method. You end up lecturing them instead of giving them time to engage in group work or you’ll end up not reaching the lesson’s objectives on time.’ (P10)

‘… In my class I have 488 students … I don’t know even half of them. I teach them in an auditorium … The desks are fixed and tiered right up preventing group work.’ (P4)

‘… Another challenge is the OBE (Outcomes-Based Education) [*system*]. All the advocated teaching strategies are good and well, but they are impossible to implement due to large group[*s*] of students, and the use of auditoria.’ (P5)

From the above statements it is evident that nurse educators realise the importance of engagement of learners with the learning content in the facilitation of meaningful learning. Bimray, Le Roux and Fakude (2013:117) confirm that facilitating a large class of first year learners using innovative pedagogical strategies has been a challenge for nurse educators. However, it can be carried out even in a class of 850 learners (Exeter *et al*. 2010:762; Wanous *et al*. 2009:79), provided nurse educators are provided with training on teaching in large classes using technology-driven and innovative pedagogical strategies (Froyd & Simpson 2008:2; Yu, Wang & Lin 2013:24). Innovative pedagogical strategies will enable learners to adapt their knowledge to solving authentic, complex, and ill-defined patients’ problems, and make rational decisions and astute clinical judgments. Lecturing to cover the content compromises learning. As learners remain passive as they do not engage with the learning content (Wulf *et al*. 2014:113). Billings and Halstead (2012:266) recommend that the lecture method is used for clarification of complex, confusing concepts, covering of background information that is not available to learners and providing information from scattered sources in order to facilitate meaningful learning. In line with the present study, Hasan (2012:2) found that the use of auditoria with fixed desks and chairs was common with large classes. However, Billings and Halstead (2012:261) recommend that in an auditorium with bolted desks, nurse educators should be creative and allow learners to sit on top of the desks to facilitate interactive small group activities.

OBE is a learner and result-centred educational approach that focuses on the instructive learning process and achievement of the desired outcomes of the learning process. It requires a democratic environment where various pedagogical strategies are utilised to achieve learning outcomes; however, learning outcomes are vaguely worded and do not focus on the core academic content (Van der Horst & McDonald 2008:5, 16). This limits the use of the intended innovative pedagogical strategies and development of higher order thinking skills, in line with the findings of this study.

When asked what can be effected in order to improve this situation, participants stated that:

‘… We should attend workshops on how to teach large classes.’ (P13)

‘… I don’t know how to facilitate some of these new teaching strategies, we should have workshops, seminars on how to use them.’ (P7)

‘… Even with OBE approach to learning that we were just told about, no one took us through the process as to what it is and how to teach using it. So I strongly feel that we need to be properly inducted into these newer teaching strategies.’ (P18)

## Difficulty in managing a large class

The study also found that managing a large class was challenging as a result of lack of active participation of learners which result in uncivil behaviour. Some participants said:

‘… In most instances, the group that is attentive are the students sitting in front, whilst the rear is more disruptive, some sleeping and others noisy.’ (P3)

‘… As a lecturer you are busy teaching, and this student is having her earphones on … listening to somebody else’s lecture because it’s better than yours. However, they are not going to utilise that information productively, because they won’t be able to integrate it with what was taught in class because they were not listening in the first place.’ (P9)

‘… Sometimes the phone rings in an auditorium, it disturbs other students … The auditorium and Murphy’s Law! The student that needs to get up and go out to answer her phone is sitting where? … In the middle of fixed chairs necessitating others to stand up!’ (P14)

‘… They are very disruptive in these large classes and this behaviour clearly indicates that they are bored.’ (P5)

These comments indicated that the nurse educators had difficulty in managing large classes because of uncivil behaviour by learners. They also had difficulty in engaging the majority of learners in their discussions as only the minority of learners in front rows were willing to engage with them. Comparable results were found by Bahanshal (2013:50). Classroom incivility is any action that interferes with a harmonious and cooperative learning environment (Feldman in Ndawo 2014:91). In the present study, learners were engaged in uncivil behaviour such as arriving late and leaving early, carrying on with off-task activities during lessons, and displaying less individual accountability. Others were engaged in increased noisey behaviour and distraction, absenteeism and disappearing, using cell phones, and sleeping in class (Clark 2008:459; Mulryan-Kyne 2010:177). In a large class, anonymity contributes significantly to uncivil behaviour and could lead in ordinary learners engaging in anti-social behaviour that display disrespect for other learners. This is disturbing, especially to the meaning- and achievement-oriented learners whose goal is to achieve high marks. Some uncivil behaviour such as letting cell phones ring display a lack of responsibility and accountability to other learners and the nurse educator. It violates the rights of learners to learn and shows disrespect to others (Ndawo 2014:93). Because of a large class, this behaviour can be repeated by many other learners if not curbed immediately. Some participants acknowledged that learners were usually engaged in uncivil behaviour because of boredom. This is confirmed by Forni (2008:16) who stated that learners become uncivil as a result of boredom, frustration and lack of stimulation in a large class. They will shift the focus to something else should the classroom situation bore them.

When they were asked about recommendations on how to deal with this situation, participants said:

‘… We need to teach them in such a way that our lectures are not boring.’ (P5)

‘… I … usually ask my colleague to accompany me. She ensures that the register is circulated and signed within class and also assists in maintaining order. Her presence allows for a conducive learning environment because it’s not easy in a large class.’ (P14)

‘… As much as they constantly chat, there are occasions when I proceed with my lesson and disregard the disruption due to time constraints.’ (P17)

### Implications for learning

From the finding of this study, large classes were experienced negatively and are not contributing to effective teaching and meaningful learning. Anonymity in a large class results in decreased motivation to learn, skipping of classes, high rates of absenteeism, and a subsequent dropout (Stickney 2008:422). In the face of a global nursing shortage, nursing cannot afford to have learners dropping out of the nursing programmes. Learners immersed in non-real-life, decontextualised and abstract learning environments are inadequately equipped with critical, reflective, creative, and innovative thinking, problem-solving, decision-making, clinical reasoning, teamwork and communication skills (Neo, Neo & Tan 2012:50). Elder *et al*. (2010:92) state that the impact of incivility to learning is that it interferes with the creation of a rich, learning environment; learners may, therefore, choose to leave the disruptive classroom, and even the learning institution, which could result in an increased dropout rate.

## Limitations of the study

The study provided rich data that nurse educators can use to improve their teaching, and maximise learners’ learning. However, the study was conducted at a nursing college in Gauteng and the findings, can, therefore, not be generalised to other nursing colleges. The study focused on the experiences of nurse educators with regard to teaching of large classes. Learners’ experiences of being taught in large classes were not explored in this context; therefore a complete picture of the teaching and learning challenges experienced cannot be conveyed.

## Recommendations

The recommendations below are from the conceptualisation of participants’ recommendations as well as from literature. The recommendations may be utilised by nurse educators in order to facilitate effective teaching and meaningful learning in learners.

### Difficulty in recognising learners as individuals in a large class

Clark (2009:194) recommends that learners should use name ‘tents so that they can be referred to by their names. They should formally introduce themselves using their full names and share any personal experience related to the course (Hasan 2012:5). Use of small groups, praising the learners’ work, an maintaining eye contact can be used to individualise learners in a large class (Elder *et al*. 2010:94). Personalised e-mail messages or short message service (SMS) texts can be sent to individual learners who are struggling academically. These messages should communicate concerns about the learner’s performance and progress. Such messages help learners feel important and cared for, therefore helping to reduce the interpersonal distance between the nurse educator and learners (Isbell & Cote 2009:185).

Nurse educators should use the didactic principle of participation and individualisation by creating interactive, thought-provoking activities that will keep more advanced learners feeling challenged and allow the less advanced learners to make progress at their own pace (Carl 2009:93; Khati 2010:1). Diagnostic pre-tests should be administered before teaching any new content in order to identify at risk learners and target remedial support for those who need extra help (Pearson & Naug 2013:137). Klein (2012:2) recommends that nurse educators should take class attendance seriously and use peer mentoring programmes. The mentoring is carried out through pairing learners who are in the first year of their nursing programme with more able learners who are in their fourth year of study to provide academic peer support. Participants in this study recommended that there should be multiple intakes of learners annually. Shields, Watson and FBiol (2008:96) state that this strategy had worked where multiple intakes were implemented and large classes divided into two; however, it resulted in an increased workload for educators, as well as increased stress. Should this strategy be used, it should be well thought through.

By 2002, four nursing colleges in Gauteng were closed because of the rationalisation process which commenced in 1996. The four remaining colleges became main nursing colleges. The learners from the closed colleges were reassigned to the main nursing colleges, thus increasing the class sizes without proportional increase in much needed resources. The increased class sizes resulted in poor realisation of the learning outcomes and lack of development of higher order thinking skills in learners (Ntsele 2008:5, 98). Currently, the DoH (2014:32) plans to reopen the closed nursing colleges as indicated in the current Strategic Plan 2014/2015 – 2018/2019.

### Difficulty in using innovative pedagogical strategies

Froyd and Simpson (2008:2) and Yu *et al*. (2013:24) recommend that nurse educators should attend seminars, workshops and conferences on teaching in large classes, using technology-driven and innovative pedagogical strategies to develop learners into self-directed, lifelong learners. Strategies recommended such as PBL, Socratic questioning, dialogic pedagogy, and social media can be used. Reasons as to why these active learning strategies are used should be communicated to learners and success reports be shown to them (McCully, Zurbrick & Ash 2014:3). Scenarios used in class must mimic real-life situations (Bimray *et al*. 2013:118). McCully *et al*. (2014:5) recommend that nurse educators should consider ‘flipping’ their classrooms by providing learning content before class, and only facilitate understanding of the same content through small group activities during class time. The inquiry and discovery-based PBL activities that are currently inconsistent with learners’ prior knowledge should be employed, as this inconsistency will motivate learners to learn (Powell & Kusuma-Powell 2011:24). Nurse educators should use the Socratic questioning method in order to stimulate learners’ critical thinking through asking probing and thought-provoking questions (Tofade, Elsner & Haines 2013:4).

Kolmos *et al*. (2008:18) and Billings and Halstead (2012:269) recommend that for PBL, nurse educators should develop realistic, comprehensive, complex, ill-defined clinical problems. Place learners in groups of 6 to 10 and assist them in signing group roles in order to facilitate interdependence. Facilitate the group(s) by questioning, probing and encouraging critical reflection, and challenge learners so that they progress satisfactorily through the problem (Bruce *et al*. 2011:202). Hong and Jacob recommend that during Socratic questioning, the following six questions are asked:

- Question of clarification ‘How does this relate to our discussion?’ Learner to state why they made a particular statement.
- Question that probe assumptions ‘What could we assume instead?’ Learner to eliminate assumptions that are not thought through.
- Question that probe reasons and evidence ‘What do you think causes that to happen? Why?’ Learner to clarify and prove that their arguments are based on facts.
- Question about viewpoints or perspective ‘What would be an alternative?’ Learner to realise that there are many perspectives to an argument.
- Question that probe implications and consequences ‘What are the consequences of that assumption?’ To bring forth the logical implications or consequences that learners can predict.
- Question about the question ‘Why do you think I asked this question?’ Learners to identify the reasons why the question was asked (Hong & Jacob 2012:18).

The process should not be rushed, and questions asked should follow on the answers given by the students. Nurse educators must ensure that the discussion is systematic and disciplined.

To facilitate dialogue in a large class, Billings and Halstead (2012:269) recommend that clear learning outcomes are communicated to learners; learning content to be discussed should be chosen based on topics of interest to learners. Hasan (2012:7) recommends organising cooperative learning activities like think-pair-share, three-step interview, and jigsaw to develop learners’ cooperative skills. Van der Horst and McDonald (2008:16) advocate that nurse educators should be properly trained and adequately supported on the use of the OBE educational approach.

Billings and Halstead (2012:415) recommend the use of social media such as Facebook; however, the instructional purposes and measurable outcomes should be communicated. Interactions using social media should be limited to the class members with use of passwords, communication posted should comply with the institution’s policies, respect for different opinions should be enforced and norms for professional behaviour should be observed. Content challenging questions can be posted and meaningful discussions facilitated.

### Difficulty in managing a large class

Vernadakis *et al*. (2012:197) recommend that learners should be challenged with real-life learning tasks that are beyond their current skill levels. Lecturing and learning tasks that are too easy produce boredom whereas learning tasks that are too difficult lead to anxiety that inhibits motivation to learn. The assistance of a colleague with the management of a large class that some participants utilised, is also recommended by Klein (2012:2). One participant stated that they consciously chose to ignore uncivil behaviour. However, Knepp (2012:41) warns that such ignorance could be interpreted by learners as educator’s assent to uncivil behaviour, hence, the behaviour could be perpetuated. Englert (2010:48) recommends the use of humour to alleviate boredom, draw the attention of learners and reduce uncivil behaviour, and to allow learners to role-play in class as these can lead to humorous situations. Clean, funny stories are a predominant form of humour used to draw the attention of students. Humour can be used effectively through use of the nursing process; assess the content that can be delivered humorously and make a presentation, diagnose the presentation for areas where emphasis is needed, formulate a plan and use resources with a desired outcome in mind and implement humour in the presentation appropriately (Englert 2010:48).

Forni (2008:21) and Knepp (2012:42) recommend a dignified proactive and preventive approach to uncivil behaviour in a large class; this should be accompanied by modelling of civil behaviour by nurse educators. Being inviting, approachable, encouraging, and make learners feel comfortable and cared for, is important. Assistance for counselling for learners who are experiencing social problems should be provided by referring them to the relevant, available resources (Goodwin & Candela 2013:616; Powell & Kusuma-Powell 2011:24). Woolfolk (2010:206) recommends that nurse educators should utilise appropriate educational principles, such as the use of striking media (e.g. colours) and motivation to reduce uncivil behaviour. Classroom rules must be established by learners so that they honour them, and should be constantly communicated to them. Bruce *et al*. (2011:177) recommend that nurse educators should adopt a value clarification approach by providing learners with opportunities to clarify and defend their values using the valuing process.

The researcher recommends that for the successful use of some of the technology-driven and innovative pedagogical strategies, nurse educators should be trained continuously in the facilitation of large classes, and use the Socratic method of questioning. A friendly, trustworthy and respectful learning environment should be maintained. Acceptance of responsibility, enthusiasm, commitment and motivation by nurse educators and learners, irrespective of the size of the class, is important (Wanous *et al*. 2009:84). Learners’ experiences of large classes should be explored, including factors that place them at high risk of academic failure.

## Conclusion

In a large class, learners feel uncared for which could result in the development of uncaring behaviour towards patients in the clinical environment. The findings in this study suggest the need for learners to feel important as unique individuals in a large class because of the impact this feeling has on the individualistic, caring and holistic nursing care that they will render to their patients in future. The need to use innovative pedagogical strategies in order to develop higher order thinking skills require a mental paradigm shift of nurse educators in order for learners to make rational decisions and astute clinical judgments for their patients.
